# Supplementary material for: The Measurement Properties and Acceptability of a New Parent–Infant Bonding Tool (‘Me and My Baby’) for Use in United Kingdom Universal Healthcare Settings: A Psychometric, Cross-Sectional Study
Source: Front Psychol. 2022 Feb 14;13:804885. doi: 10.3389/fpsyg.2022.804885 (PMC8883030; doi:10.3389/fpsyg.2022.804885)

**Supplementary Material for “The measurement properties and acceptability of a new parent-infant bonding tool (‘Me and My Baby’) designed for use in United Kingdom universal healthcare settings: A psychometric, cross-sectional, study”**

**Measure S1.** Me and My Baby. NOTE: this measure is under further development. Please contact the corresponding author if you wish to use it in the format below.

**Me and My Baby**

Having a new baby can bring up lots of different feelings and emotions. This questionnaire is designed to explore how you are feeling about being a parent to your baby.

Answering these questions will help us to understand how things are going for you. There is space on the back of this page for you and your Health Visitor to explore why you have answered the way you have if you wish. **Thinking about your feelings about your baby, choose the response for each statement that feels right to you…**

**
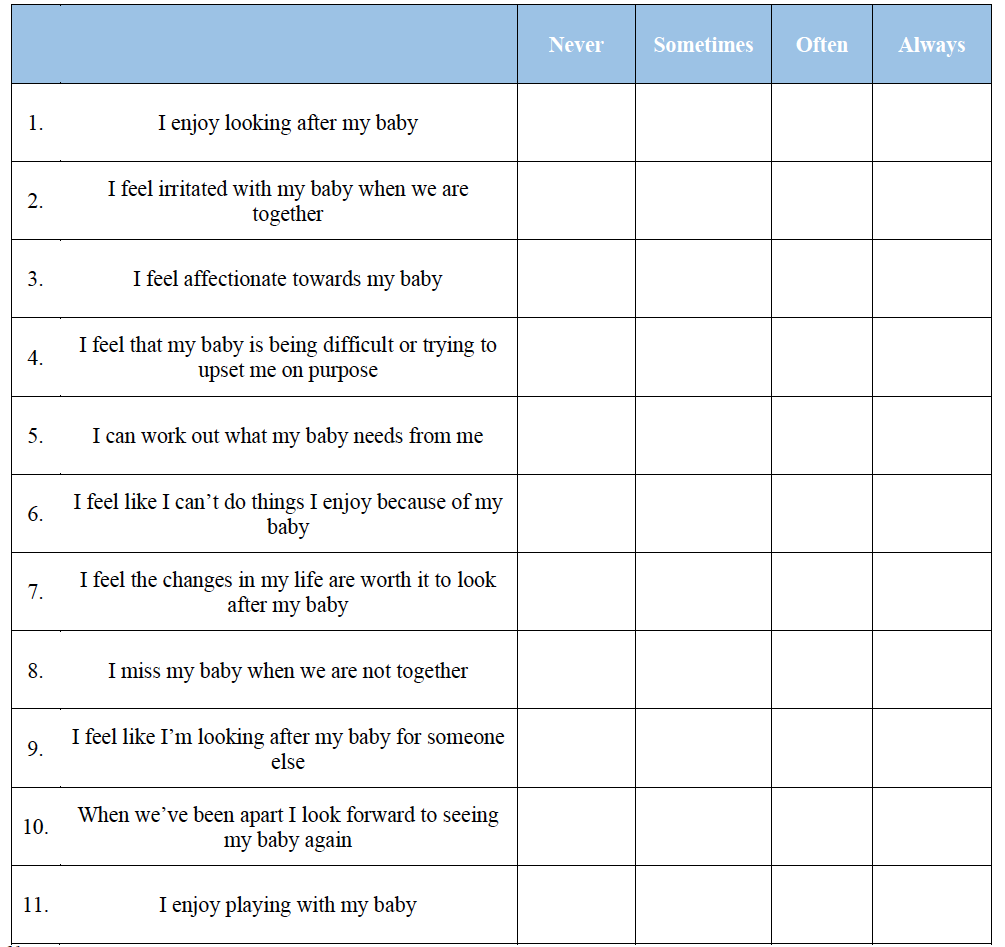
**

**
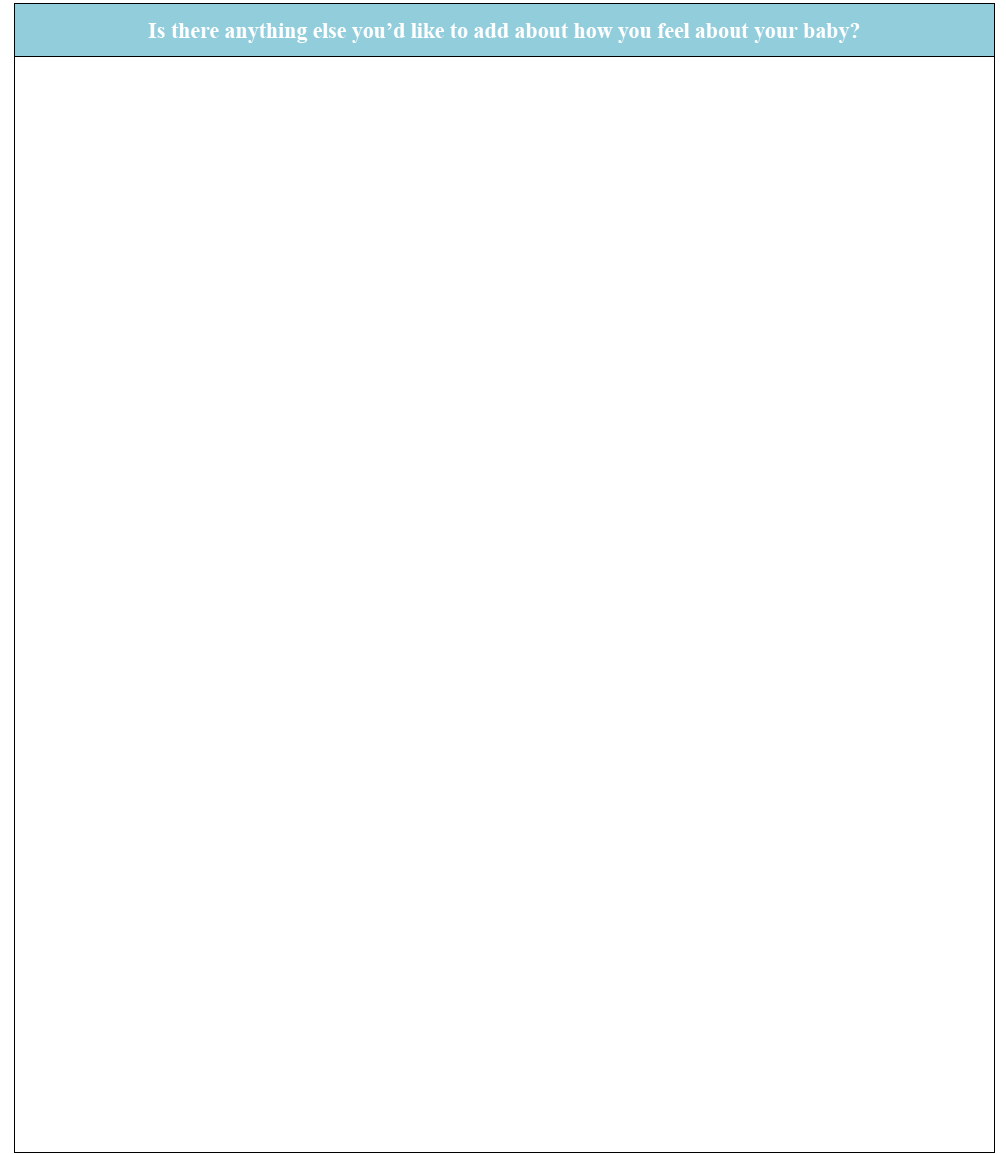
**

**Scoring Sheet:** The score for each response is in the equivalent box below – find the option selected by the parent for each question and add up the scores. Higher scores on this tool suggest that a parent is finding it difficult to develop an appropriate bond with their infant. **It is important to note that there are no validated cut offs for clinical concern on this tool – so combine scores with your professional judgement when deciding what to do next for a parent.**


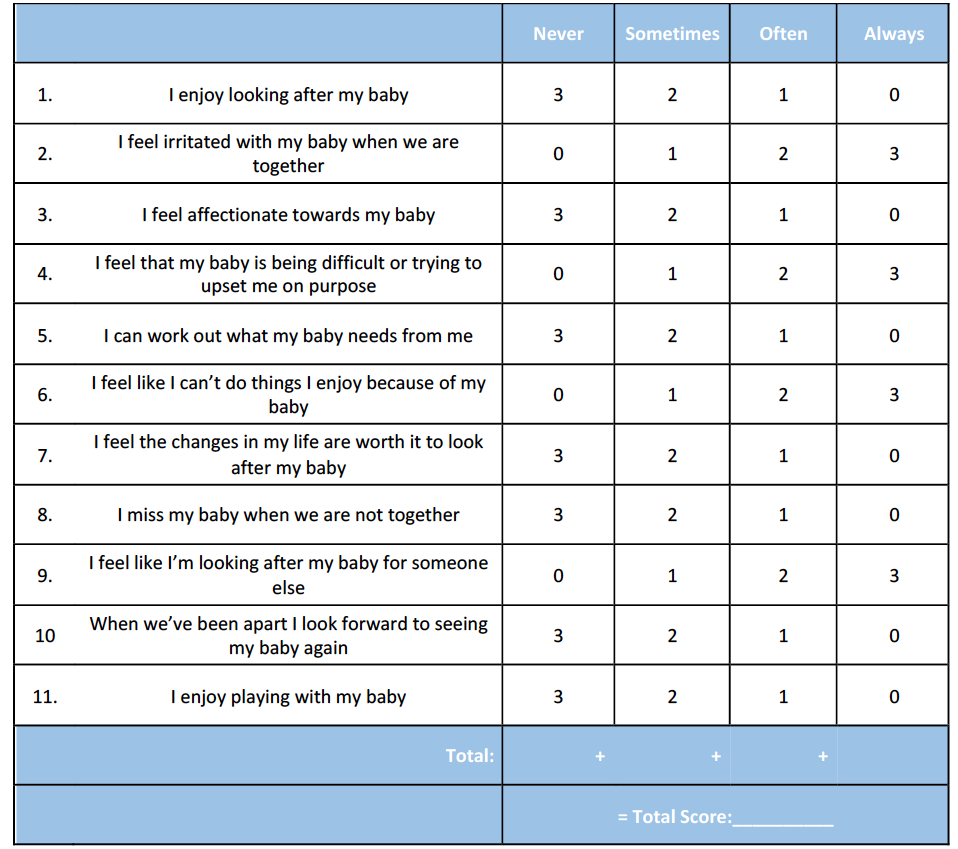


**Information Sheet S1.**

**(To include logos)**

**Information on the ‘Me and My Baby’ questionnaire**

**What will happen at your next Health Visitor visit?**

When your baby is between 6 and 8 weeks old your Health Visitor will talk to you about how you and your baby are getting along. At this visit your Health Visitor will ask if you have any questions about the information in this leaflet, and if you’re willing to complete some questions about your relationship with your baby.

**Why are you asking about my relationship with my baby?**

The Me and My Baby questionnaire is short and has been developed by Health Visitors and researchers, with other NHS staff, and with input from parents.

Not everyone finds it easy to get on with their new baby. Some mums, even if they have other children, sometimes feel they don’t understand their new baby, or that their baby is being difficult on purpose.

Also, when things are going well, many mums find it useful to reflect on their feelings about their baby. If you feel like things aren’t going how you want them to your Health Visitor can help you. We are asking all mums in your area to complete the questionnaire. For now, we are only asking biological mums who are the main carers of their new baby.

**Why are you asking these questions?**

In partnership with the Department of Health Sciences at the University of York, we are exploring how useful these questions are in showing when relationships between mum and baby are going well and not so well. You don’t have to answer these questions if you don’t want to, and you can stop completing the questionnaire at any time – your decision will not affect your relationship with your Health Visitor or the support they offer you.

**What will happen to my answers?**

Your Health Visitor will look at your answers and talk to you about your relationship with your baby. Health Visitors are highly trained and understand that being a mum is different for everyone.

If the questionnaire is useful, it may help Health Visitors in offering future support and training to parents around forming a good relationship with their baby. Your answers will be shared with colleagues in the Department of Health Sciences at The University of York (the university are partnering with Rotherham Doncaster and South Humber NHS Foundation Trust to explore the usefulness of the questionnaire).

### How will we use information about you?

### Your NHS Trust will not share any identifiable information about you (e.g. your name or address) with the University of York. The University will examine all anonymous answers on the Me and My Baby questionnaire to see if the questions are helpful in identifying where the relationship between mums and their new baby may be difficult or where some support may be helpful. These findings could help to improve the care new mums across your area receive in the future. The findings will be shared in reports, copies of which will be available on the following websites:

- If you live in xxxxx see xxxxx
- If you live in xxxxx see xxxxx
- Research team website: https://www.arc-yh.nihr.ac.uk/home

The research team at the University of York will only have access to fully anonymised data, they will not receive any data or codes that can be used to identify you and they will not be able to see your name or contact details.

The research team will keep all data safe and secure on University of York servers. Once we have finished the study, the University of York will keep the fully anonymised data for 10 years at which point it will be securely destroyed.

### What are your choices about how your information is used?

- You can stop being part of the study at any time, without giving a reason, but we will keep information about you that we already have.
- We need to manage your records in specific ways for the research to be reliable. This means that we won’t be able to let you see or change the data we hold about you.

### Where can you find out more about how your information is used?

*You can find out more about how we use your information*

- *at* [*www.hra.nhs.uk/information-about-patients/*](about:blank)
- *by asking a member of the research team* ***sarah.blower@york.ac.uk***
- The sponsor for this study is the University of York [https://www.york.ac.uk/staff/research/governance/research-policies/policy-for-clinical-research](about:blank)
- at the University of York data protection officer’s website: [https://www.york.ac.uk/records-management/dp/](about:blank)
- *by ringing your Health visiting service on the numbers below*

**If you would like more information, please contact your Health Visiting service in XXXXX on XXXXX, or XXXXX on XXXX**

**Table S1.** Standardised covariance matrix (polychoric correlations) as estimated from an ordinal factor analysis of the items of the MaMB scale, using the FACTOR software package.

| **MamB item number and abbreviated wording** | | **1** | **2** | **3** | **4** | **5** | **6** | **7** | **8** | **9** | **10** | **11** |
| --- | --- | --- | --- | --- | --- | --- | --- | --- | --- | --- | --- | --- |
| **1** | Enjoy looking after baby | 1.00 |  |  |  |  |  |  |  |  |  |  |
| **2** | Feel irritated with baby | 0.65 | 1.00 |  |  |  |  |  |  |  |  |  |
| **3** | Affectionate towards baby | 0.74 | 0.58 | 1.00 |  |  |  |  |  |  |  |  |
| **4** | Feel baby is being difficult | 0.62 | 0.53 | 0.67 | 1.00 |  |  |  |  |  |  |  |
| **5** | Can work out baby’s needs | 0.43 | 0.49 | 0.38 | 0.42 | 1.00 |  |  |  |  |  |  |
| **6** | Can’t do enjoyable things because of baby | 0.57 | 0.59 | 0.47 | 0.51 | 0.39 | 1.00 |  |  |  |  |  |
| **7** | Life changes worth it | 0.59 | 0.44 | 0.56 | 0.35 | 0.28 | 0.40 | 1.00 |  |  |  |  |
| **8** | I miss my baby when not together | 0.60 | 0.49 | 0.64 | 0.35 | 0.27 | 0.43 | 0.45 | 1.00 |  |  |  |
| **9** | Feels like someone else’s baby | 0.46 | 0.44 | 0.51 | 0.44 | 0.34 | 0.35 | 0.44 | 0.43 | 1.00 |  |  |
| **10** | Look forward to seeing baby again | 0.63 | 0.41 | 0.61 | 0.28 | 0.38 | 0.41 | 0.41 | 0.76 | 0.57 | 1.00 |  |
| **11** | Enjoy playing with | 0.64 | 0.46 | 0.74 | 0.55 | 0.33 | 0.45 | 0.55 | 0.56 | 0.58 | 0.66 | 1.00 |

**Figure S1**. Category probability plots for the 11 items of the MaMB scale.


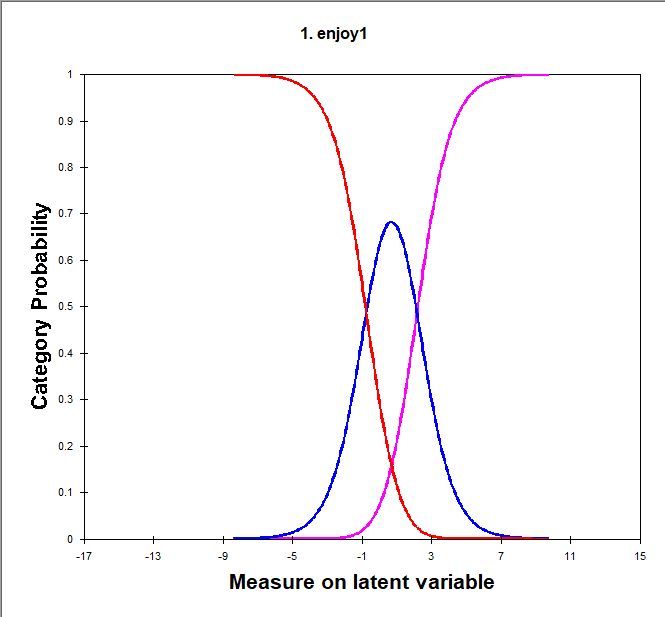

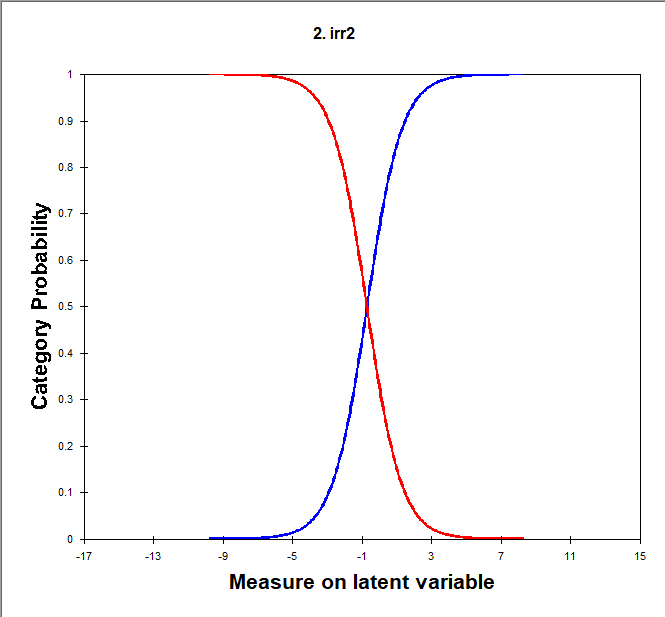

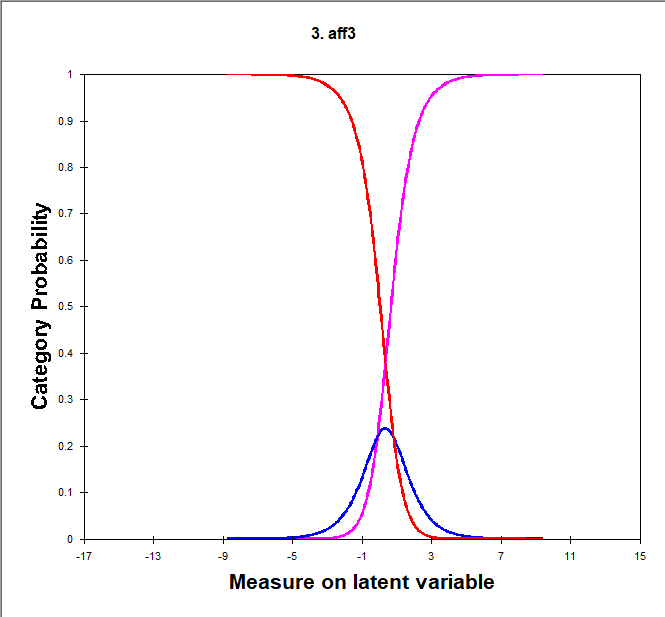

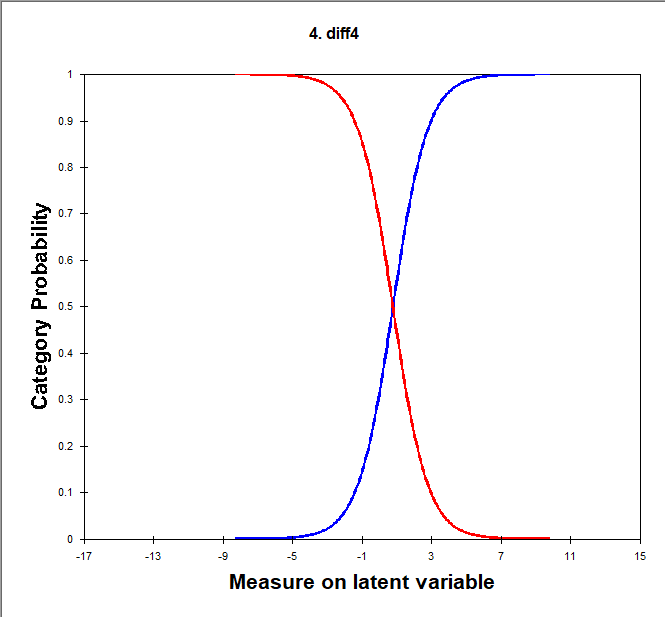


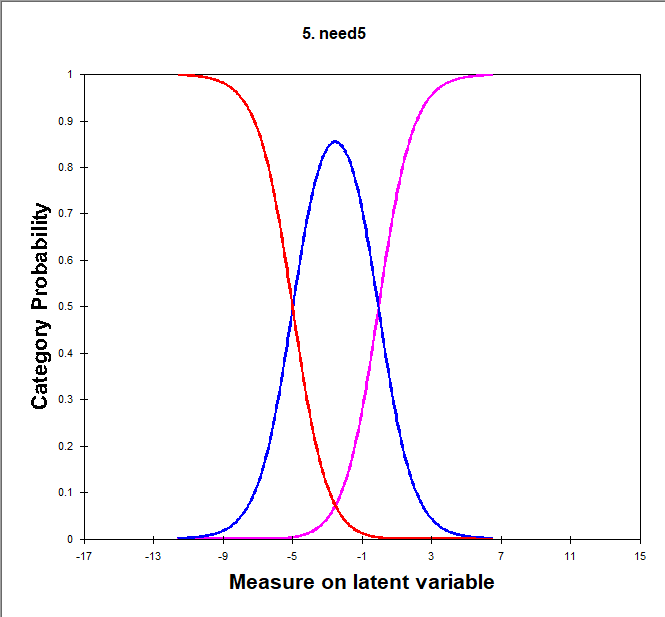

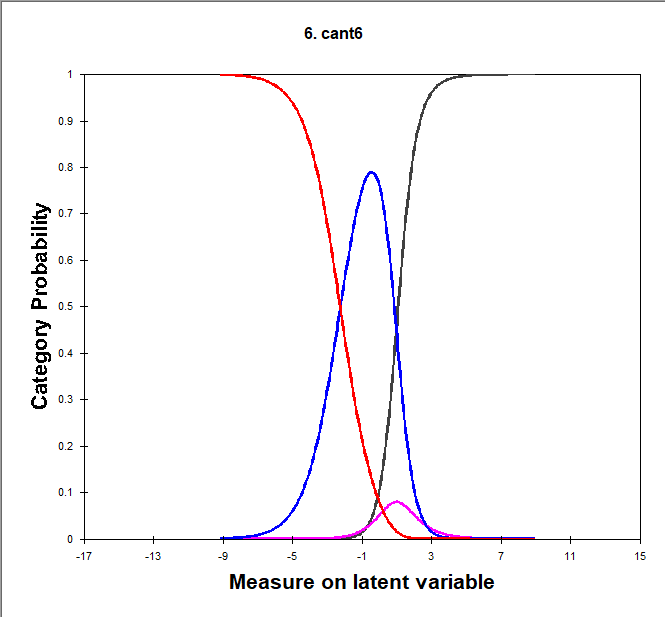


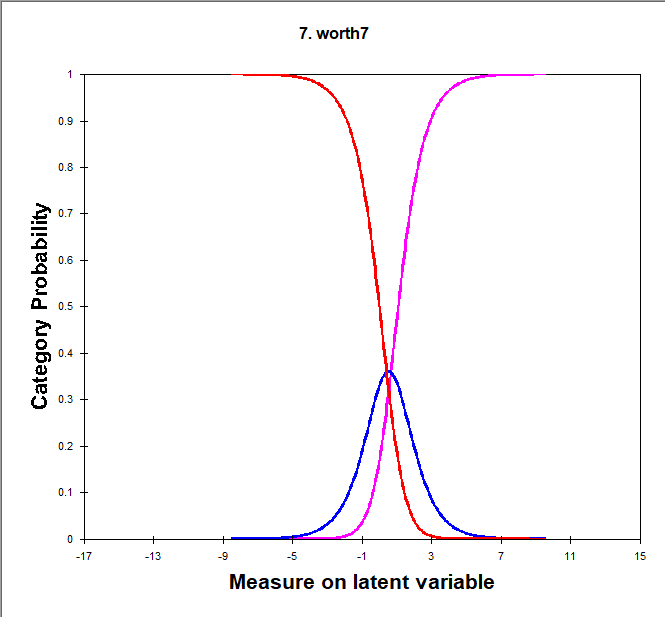

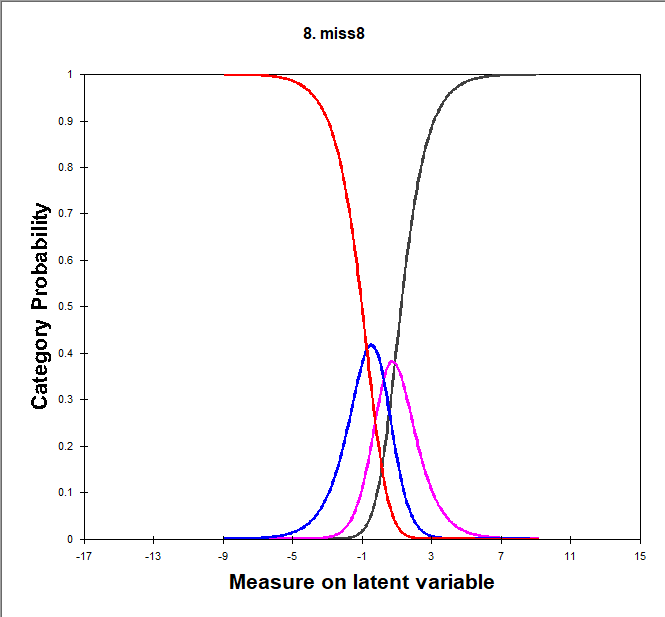


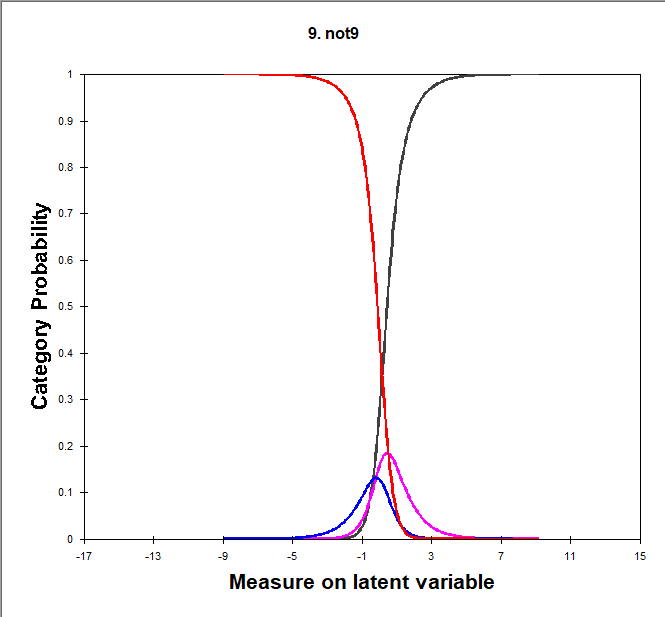

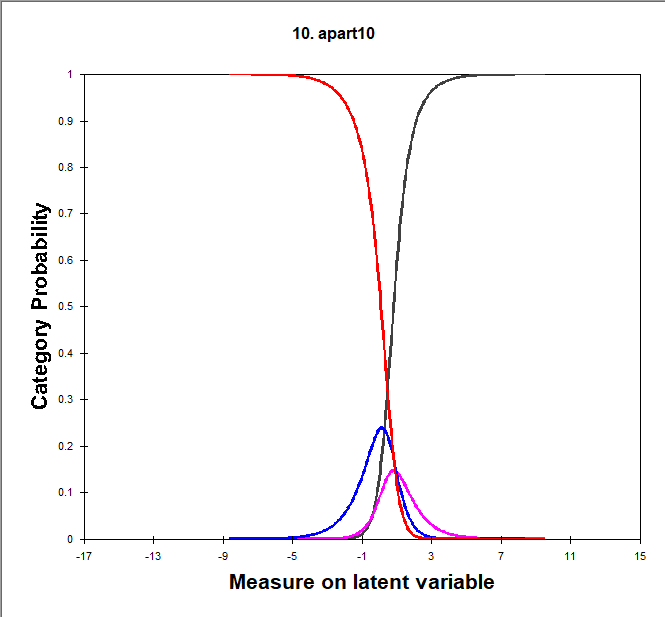


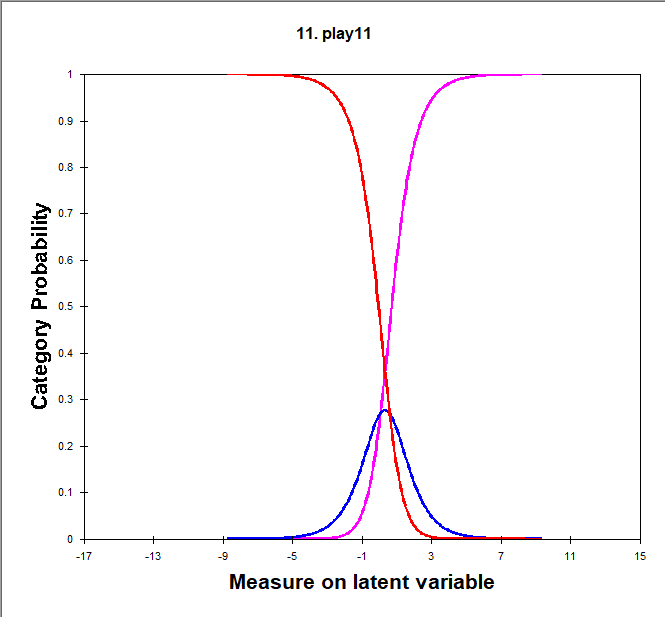


Note: that in Figure S1 Winsteps treats items with unobserved response categories as trichotomous or dichotomous items, and generates the response category probability curves accordingly.

**Figure S2.** Test Information curve for the MaMB questionnaire. Note, in this case the latent variable is conceptualised as ‘difficulties with bonding’.


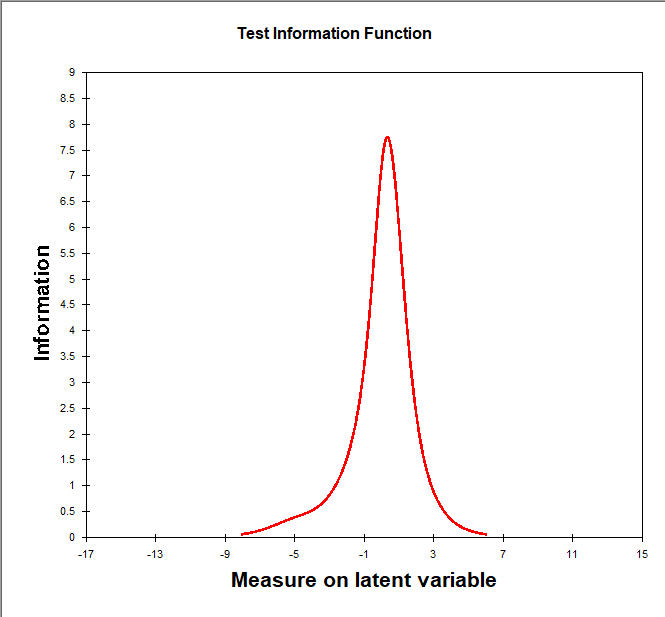

Supplement: Supplementary file 1 [file Data_Sheet_1.docx]
